# Catheter Ablation vs. Anti-Arrhythmic Drugs as First-Line Treatment in Symptomatic Paroxysmal Atrial Fibrillation: A Systematic Review and Meta-Analysis of Randomized Clinical Trials

**DOI:** 10.3389/fcvm.2021.664647

**Published:** 2021-05-21

**Authors:** Andrea Saglietto, Fiorenzo Gaita, Roberto De Ponti, Gaetano Maria De Ferrari, Matteo Anselmino

**Affiliations:** ^1^Division of Cardiology, Department of Medical Sciences, “Città della Salute e della Scienza di Torino” Hospital, University of Turin, Turin, Italy; ^2^Cardiology Unit, J Medical, Turin, Italy; ^3^Department of Medicine and Surgery, University of Insubria, Varese, Italy

**Keywords:** atrial fibrillation, catheter ablation, rhythm control, anti-arrhythmic drugs, side effects

## Abstract

**Background:** Catheter ablation has become a well-established indication for long-term rhythm control in atrial fibrillation (AF) patients refractory to anti-arrhythmic drugs (AADs). Efficacy and safety of AF catheter ablation (AFCA) before AADs failure are, instead, questioned.

**Objective:** The aim of the study was to perform a systematic review and meta-analysis of randomized clinical trials (RCTs) comparing first-line AFCA with AADs in symptomatic patients with paroxysmal AF.

**Methods:** We performed a random-effects meta-analysis of binary outcome events comparing AFCA with AADs in rhythm control-naïve patients. The primary outcomes, also stratified by the type of ablation energy (radiofrequency or cryoenergy), were (1) recurrence of atrial tachyarrhythmias and (2) recurrence of symptomatic atrial tachyarrhythmias. The secondary outcomes included adverse events.

**Results:** Six RCTs were included in the analysis. AFCA was associated with lower recurrences of atrial tachyarrhythmias [relative risk (RR) 0.58, 95% confidence interval (CI) 0.46–0.72], consistent across the two types of ablation energy (radiofrequency, RR 0.50, 95% CI 0.28–0.89; cryoenergy, RR 0.60, 95% CI 0.50–0.72; *p*-value for subgroup differences: 0.55). Similarly, AFCA was related to less symptomatic arrhythmic recurrences (RR 0.46, 95% CI 0.27–0.79). Overall, adverse events did not differ. A trend toward increased periprocedural cardiac tamponade or phrenic nerve palsy was observed in the AFCA group, while more atrial flutter episodes with 1:1 atrioventricular conduction and syncopal events were reported in the AAD group.

**Conclusions:** First-line rhythm control therapy with AFCA, independent from the adopted energy source (radiofrequency or cryoenergy), reduces long-term arrhythmic recurrences in patients with symptomatic paroxysmal AF compared with AADs.

## Introduction

Atrial fibrillation (AF) is the most common supraventricular arrhythmia, affecting up to 2% of the population (1). Anti-arrhythmic drugs (AADs) are considered as the first-line option for the maintenance of sinus rhythm (rhythm control) in patients with symptomatic AF episodes; however, they are limited by a relatively low efficacy and substantial side effects (2–4). AF catheter ablation (AFCA) has established itself as a superior alternative to AADs in terms of long-term sinus rhythm maintenance and quality of life (5, 6). Accordingly, AFCA has been recommended in case of AAD failure by the most recent guidelines (1, 7).

A possible role for AFCA also as first-line option in paroxysmal AF is emerging. A shorter time between first AF documentation and ablation is associated with lower arrhythmic recurrences, implying a lower probability of disease progression toward persistent AF (4, 8, 9). In the last decade, three randomized clinical trials (RCTs) evaluating radiofrequency (RF) ablation compared with AADs as first-line treatment in rhythm control-naïve patients (10–12) had demonstrated lower recurrence rate by ablation, at the price of transiently exposing the patient to the rare, but not negligible, risk of periprocedural complications (13). More recently, three RCTs comparing cryoballoon ablation and AADs as first-line rhythm control therapy have been published (14–16).

The aim of the present study is to perform an updated systematic review and meta-analysis comparing efficacy and safety of AFCA vs. AADs as first-line rhythm control strategy in patients with paroxysmal AF, also assessing potential differences related to ablation energy source (RF or cryoenergy).

## Methods

The present systematic review and meta-analysis was performed in accordance to Preferred Reporting Items for Systematic Reviews and Meta-Analyses (PRISMA) guidelines (17).

### Search Strategy, Study Selection, and Quality Assessment

We screened PubMed/MEDLINE and Embase databases from their inceptions to March 7, 2021, using the following search terms: “atrial fibrillation AND ablation AND first-line.”

The inclusion criteria were as follows: (a) prospective RCTs comparing AFCA (RF or cryoballoon ablation) with AADs as first-line rhythm control treatment in symptomatic AF; and (b) availability of data regarding arrhythmic recurrences (symptomatic or asymptomatic).

Two investigators (AS and MA) independently reviewed the titles/abstracts and studies to determine their eligibility based on the inclusion criteria and extracted all the relevant features, including study characteristics, baseline population data, and outcomes (assessment and measures). A third reviewer (GMDF) resolved disagreement. Risk of bias assessment was performed at the study level using the Cochrane bias risk assessment tool (RoB 2) (18).

### Outcomes

The primary efficacy outcomes of the present analysis were as follows: (1) recurrence of atrial tachyarrhythmias and (2) recurrence of symptomatic atrial tachyarrhythmias. According to study design, recurrent atrial tachyarrhythmia included AF alone, or AF and/or atrial tachycardia/atrial flutter (AT/AFL).

The secondary outcome endpoints were all-cause death, proportion of crossover to the alternative arm, proportion of patients undergoing ablation after failure of the initial treatment (either AFCA or AADs), stroke/transient ischemic attack (TIA), cardiac tamponade, phrenic nerve palsy, atrioesophageal fistula, pulmonary vein (PV) stenosis >70%, atrial flutter with 1:1 AV conduction, ventricular tachycardia, symptomatic bradycardia requiring pacemaker implantation, and syncope.

### Statistical Analysis

Baseline characteristics of pooled study populations were reported as median values between the included studies, along with their interquartile range (IQR). Random-effects model meta-analysis of binary outcome events, taking into account the estimated between-study heterogeneity, was performed for the present analysis using inverse-variance method. The resulting meta-analytic relative risk (RR) of observing the investigated outcomes in AFCA group compared with AAD group, along with their 95% confidence interval (CI), is reported. Forest plots for the primary outcome endpoints are shown, stratified by ablation energy type (RF or cryoenergy). A test for subgroup differences was also performed. Heterogeneity across studies was assessed using the Cochran Q test. Higgins I^2^ statistics was used to determine the degree of between-study heterogeneity (I^2^ <25%, low; 25–50%, moderate; and >50%, high degree of heterogeneity). Due to the low number of studies included, we did not investigate publication bias, and we did not performed meta-regression to assess potential source of heterogeneity. *P*-values < 0.05 were considered statistically significant. Statistical analyses were performed with R version 4.0.0 (R Foundation for Statistical Computing, Vienna, Austria).

## Results

### Included Studies and Population Characteristics

**Figure 1:**
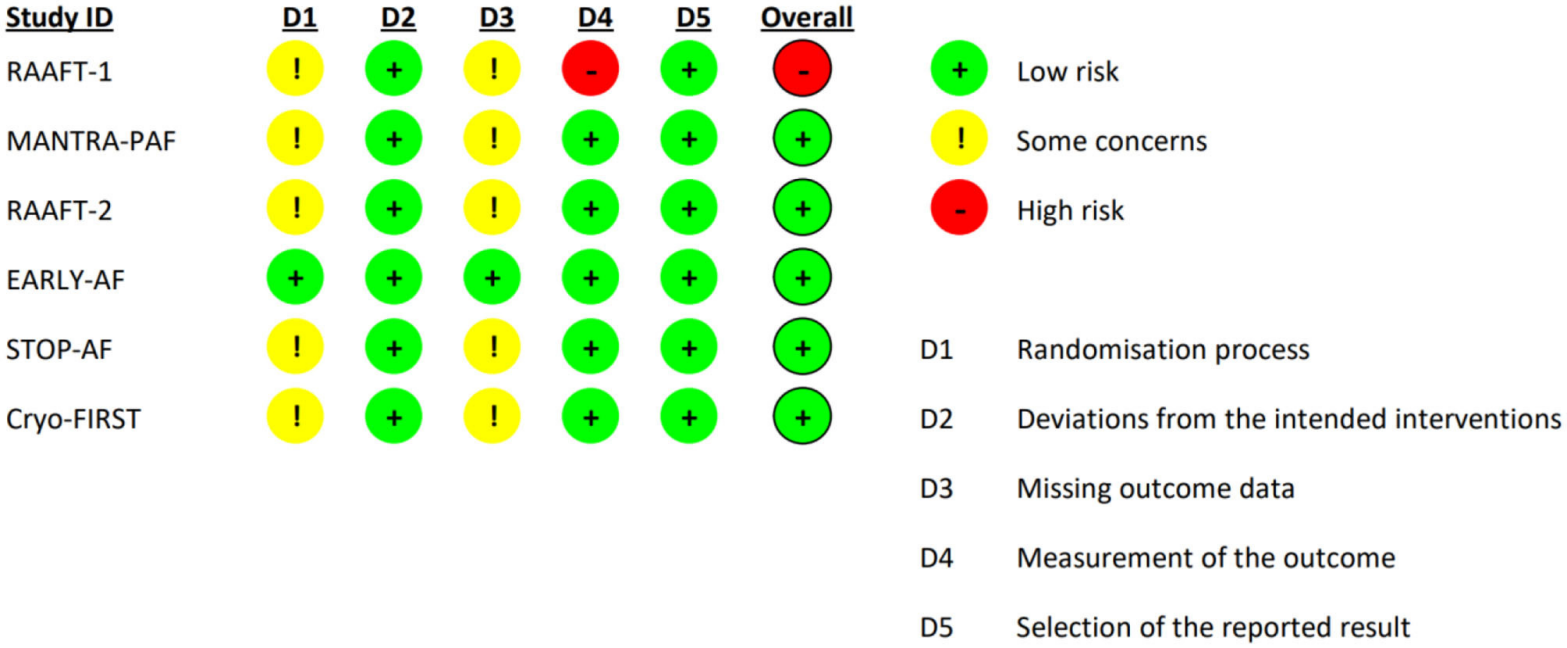
Risk of bias assessment using Cochrane risk of bias 2 (RoB 2) tool.

A total of 342 studies were identified by literature search. After detailed evaluation, six RCTs (10–12, 14–16) were finally included in the systematic review and meta-analysis (refer to Supplementary Figure 1 for PRISMA flowchart). The 5-year follow-up data of MANTRA-PAF (19) was not considered since clinical assessment and electrocardiographic monitoring were not performed between the second and 5th year. The main characteristics of the included RCTs are reported in Table 1. Ablation energy was RF in RAAFT-1 (10), MANTRA-PAF (11), and RAAFT-2 (12) while cryoenergy in EARLY-AF (14), STOP-AF (15), and Cryo-FIRST (16). Further, details regarding study inclusion/exclusion criteria and study-specific endpoints may be found in the Supplementary Tables 1, 2. Risk of bias in the individual studies is reported in Figure 1: the overall risk of bias for five studies was low, while RAAFT-1 trial (10) was considered at high risk of bias due to the unblinded outcome adjudication.

**Table 1:** Main characteristics of the included randomized clinical trials comparing first-line catheter ablation with antiarrhythmic drugs in symptomatic atrial fibrillation.

**Study**	**Study type**	**Enrollment period**	**No. of patients included in the primary analysis (AFCA/AADs)**	**Paroxysmal AF (%)**	**Type of ablation energy used**	**Follow-up duration (months)**
RAAFT-1 [Wazni et al. (10)]	Prospective, randomized, multicenter	2001–2002	32/35	96%	Radiofrequency	12
MANTRA-PAF [Cosedis Nielsen et al. (11)]	Prospective, randomized, multicenter	2005–2009	146/148	100%	Radiofrequency	24
RAAFT-2 [Morillo et al. (12)]	Prospective, randomized, multicenter	2006–2010	66/61	98%	Radiofrequency	24
EARLY-AF [Andrade et al. (14)]	Prospective, randomized, multicenter	2017–2018	154/149	95%	Cryoenergy	12
STOP-AF [Wazni et al. (15)]	Prospective, randomized, multicenter (Canada)	2017–2019	104/99	100%	Cryoenergy	12
Cryo-FIRST [Kuniss et al. (16)]	Prospective, randomized, multicenter	2014–2018	107/111	100%	Cryoenergy	12

*AFCA, atrial fibrillation catheter ablation; AADs, anti-arrhythmic drugs; AF, atrial fibrillation*.

The resulting meta-analytic population encompassed 1,204 patients [603 randomized to AFCA (365 cryoballoon ablation, 61%; 238 RF ablation, 39%) and 601 to AADs], with a median follow-up of 12 (IQR 12–24) months. AF type was paroxysmal in nearly the totality of the patients (99%, IQR 96–100%). Median age was 55 (IQR 54–59) years, with nearly 2:1 male-to-female ratio (male 70%, IQR 68–71%). Hypertension was the most frequent concurrent comorbid condition (37%, IQR 33–41%), while baseline heart failure was rare [2%, IQR 1–6%; left ventricular ejection fraction (LVEF) 61%, IQR 60–61%]. Median left atrial antero-posterior diameter was 40 mm (IQR 38–41 mm). Mean CHADS_2_ score in MANTRA-PAF (11) and RAAFT-2 (12) was 0.6, while mean CHA_2_DS_2_-VASc score was 1.9 in EARLY-AF (14); 66% and 86% of the patients in STOP-AF (15) and in Cryo-FIRST (16), respectively, had a CHA_2_DS_2_-VASc score ≤ 2; and 58% (IQR 51–60%) of the patients were on beta-blocker therapy at the time of randomization.

Table 2 reports study-specific ablation protocol, AAD use in both treatment arms, type of monitoring, and definition of arrhythmic recurrence. All patients underwent PV isolation (PVI). In two RF studies, additional ablation lesion was also allowed at the physician's discretion [MANTRA-PAF (11) and RAAFT-2 (12)], while in Cryo-FIRST study, additional freeze applications were allowed in case of incomplete PVI isolation or focal trigger identification (16). AAD use in AFCA arm was only allowed during post-procedural blanking period in most of the studies. Continuous electrocardiographic monitoring with an implantable loop recorder was only performed in EARLY-AF (14).

**Table 2:** Study-specific ablation protocol, AAD use in both treatment arms, type of monitoring, and definition of arrhythmic recurrence.

**Study**	**Ablation protocol**	**AAD therapy**	**AAD use after index ablation in AFCA arm**	**Arrhythmic recurrence monitoring**	**Definition of arrhythmic recurrence**
RAAFT-1 [Wazni et al. (10)]	PVI	Flecainide, propafenone, and sotalol (according to physician preference)	Not permitted	Clinical follow-up and intermittent electrocardiographic monitoring (event recorder, 24-h Holter)	Any recurrence of AF lasting longer than 15 s
MANTRA-PAF [Cosedis Nielsen et al. (11)]	PVI + additional lesions allowed (according to physician preference)	Propafenone, flecainide, sotalol, dofetilide, and amiodarone (according to physician preference)	Only allowed during the 90-day blanking period	Clinical follow-up and intermittent electrocardiographic monitoring (7-day Holter)	Any recurrence of AF lasting longer than 1 min
RAAFT-2 [Morillo et al. (12)]	PVI + additional lesions allowed (according to physician preference)	Flecainide, propafenone, sotalol, and amiodarone (according to physician preference)	Only allowed during the 3-month blanking period	Clinical follow-up and intermittent electrocardiographic monitoring (event recorder, 24-h Holter)	Any recurrence of atrial tachyarrhythmia (AF, AT/AFL) lasting longer than 30 s
EARLY-AF [Andrade et al. (14)]	PVI	Flecainide, propafenone, dronedarone, sotalol, and amiodarone (according to physician preference)	Only allowed during the blanking period (excluding amiodarone); to be discontinued at least five half-lives before the end of the 90-day blanking period	Clinical follow-up and continuous electrocardiographic monitoring (loop recorder)	Any recurrence of atrial tachyarrhythmia (AF, AT/AFL) lasting longer than 30 s
STOP-AF [Wazni et al. (15)]	PVI	Flecainide, propafenone, dronedarone, sotalol, and amiodarone (according to physician preference)	Only allowed for up to 80 days after the procedure (excluding amiodarone), to allow complete washout by the end of the 90-day blanking period	Clinical follow-up and intermittent electrocardiographic monitoring (event recorder, 24-h Holter)	Any recurrence of atrial tachyarrhythmia (AF, AT/AFL) lasting longer than 30 s
Cryo-FIRST [Kuniss et al. (16)]	PVI + additional freeze applications allowed (in case of incomplete PV isolation or focal trigger identification)	Flecainide, propafenone, dronedarone, sotalol, and amiodarone (according to physician preference)	Only allowed during the 3-month blanking period	Clinical follow-up and intermittent electrocardiographic monitoring (7-day Holter)	Any recurrence of atrial tachyarrhythmia (AF, AT/AFL) lasting longer than 30 s

*AAD, antiarrhythmic drug; AFCA, atrial fibrillation catheter ablation; AF, atrial fibrillation; PVI, pulmonary vein isolation; AT/AFL, atrial tachycardia/atrial flutter*.

### Primary Endpoints

The threshold defining an arrhythmic recurrence varied between 15 s in RAAFT-1 (10) and 60 s in MANTRA-PAF (11), with the four remaining studies used a 30-s threshold (12, 14–16).

**Figure 2:**
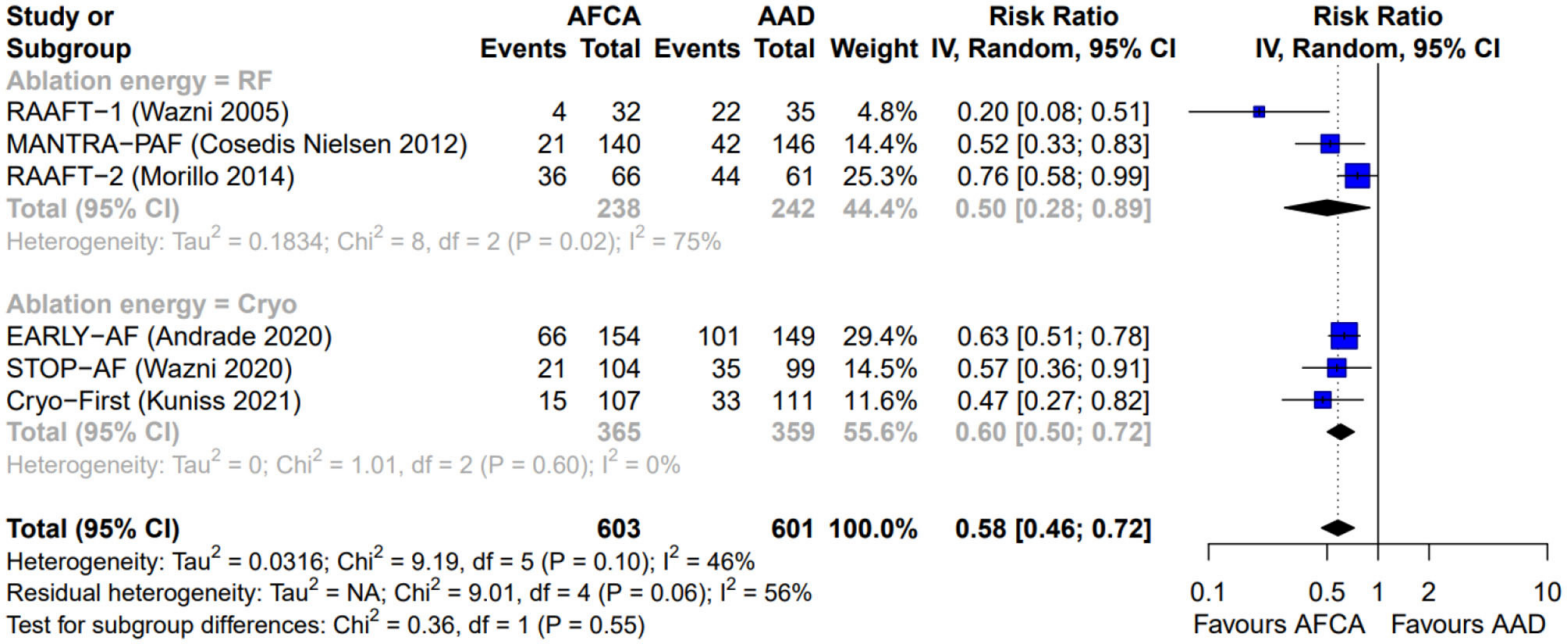
Forest plot reporting the risk of recurrence of atrial tachyarrhythmias, stratified by ablation energy (radiofrequency or cryoenergy).

**Figure 3:**
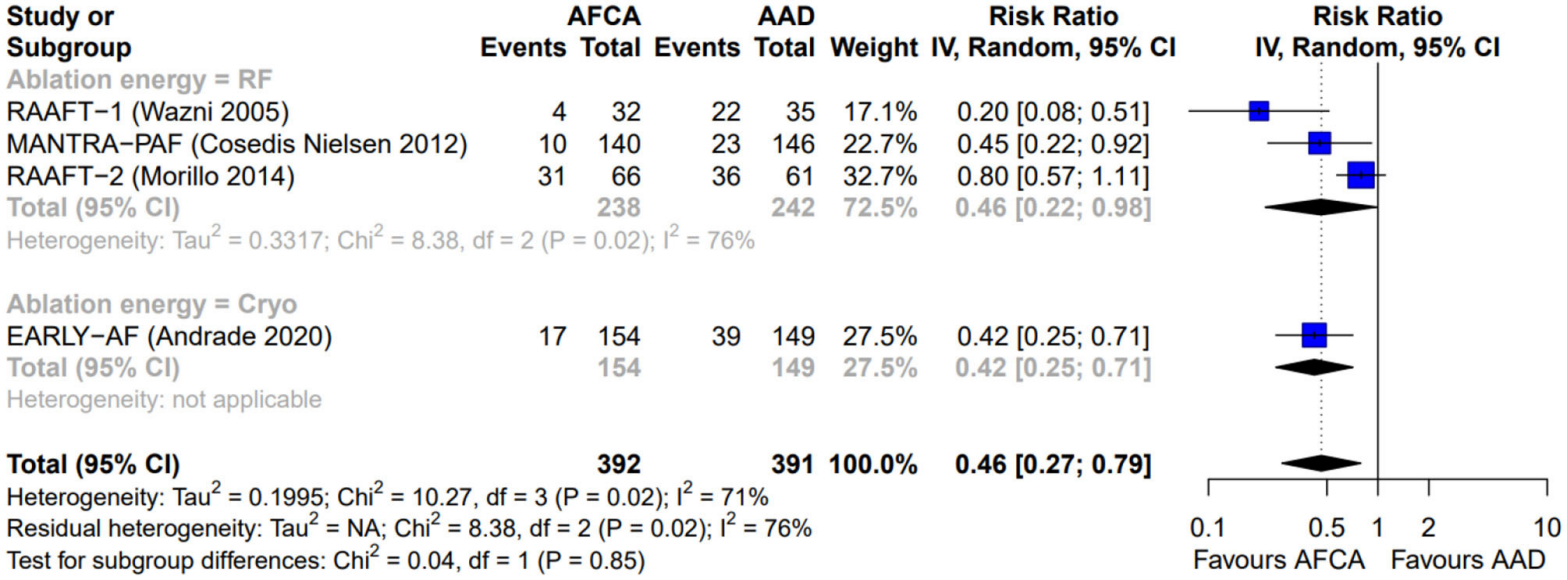
Forest plot reporting the risk of recurrence of symptomatic atrial tachyarrhythmias, stratified by ablation energy (radiofrequency or cryoenergy).

As illustrated in Figure 2, AFCA was associated with a higher probability of freedom from any arrhythmic recurrence compared with AADs (RR 0.58, 95% CI 0.46–0.72, I^2^ = 46%), consistent across the two types of ablation energy used (RF, RR 0.50, 95% CI 0.28–0.89, I^2^ = 75%; cryoenergy, RR 0.60, 95% CI 0.50–0.72, I^2^ = 0%; *p*-value for subgroup differences: 0.55). Similarly, AFCA is related to higher probability of freedom from symptomatic arrhythmic recurrence (Figure 3; RR 0.46, 95% CI 0.27–0.79, I^2^ = 71%), irrespective of ablation energy used (RF, RR 0.46, 95% CI 0.22–0.98, I^2^ = 76%; cryoenergy, RR 0.42, 95% CI 0.25–0.71, I^2^ = 0%; *p*-value for subgroup differences: 0.85). Sensitivity analysis only considering studies with low risk of bias gave similar results (Supplementary Figures 2, 3).

### Secondary Endpoints

Secondary endpoints are detailed in Table 3, while forest plots are reported in Supplementary Figures 4–14. All-cause deaths did not differ between AFCA and AAD groups (0.5 vs. 0.7%, RR 0.86, 95% CI 0.28–2.67, I^2^ = 0%). Crossover to the alternative arm was significantly less frequent in AFCA compared with AAD group (7.9 vs. 28.2%; RR 0.23, 95% CI 0.10–0.51; I^2^ = 77%). No significant differences in terms of stroke/TIA (0.8 vs. 0.8%, RR 0.98, CI 0.33–2.95; I^2^ = 0%) were found. Concerning procedural complications, there was a nons-ignificant trend toward more cardiac tamponade/clinically significant pericardial effusion (1.3 vs. 0.2%; RR 2.35, CI 0.62–8.93; I^2^ = 0%) or phrenic nerve palsy (1.4 vs. 0.3%; RR 2.42, CI 0.40–14.63; I^2^ = 4%) in the AFCA group. No atrio-esophageal fistula were observed, and only one severe PV stenosis was documented after an RF ablation procedure. Conversely, a trend toward more AFL episodes with 1:1 atrioventricular conduction (0 vs. 1.5%; RR 0.25, CI 0.03–2.25; I^2^ = 0%) and syncope (0.2 vs. 1.4%; RR 0.30, CI 0.08–1.12; I^2^ = 0%) was observed in the AAD group.

**Table 3:** Meta-analysis results for pre-specified secondary outcomes.

**Secondary outcomes**	**No. of studies**	**Included patients (AFCA/AAD)**	**AFCA (% of included patients)**	**AADs (% of included patients)**	**RR (95% CI)**
All-cause death	6	1204 (603/601)	3 (0.5%)	4 (0.7%)	0.86 (0.28–2.67)
Crossover to alternative treatment arm	6	1204 (603/601)	48 (7.9%)	169 (28.2%)	0.23 (0.10–0.51)
Ablation during follow-up	6	1204 (603/601)	96 (15.9%)	169 (28.2%)	0.33 (0.12–0.88)
Stroke or transient ischemic attack	6	1204 (603/601)	5 (0.8%)	5 (0.8%)	0.98 (0.33–2.95)
Cardiac tamponade or clinically significant pericardial effusion	6	1204 (603/601)	8 (1.3%)	1 (0.2%)	2.35 (0.62–8.93)
Phrenic nerve palsy	3^*^	724 (365/359)	5^§^ (1.4%)	1^§^ (0.3%)	2.42 (0.40–14.63)
Atrioesophageal fistula	6	1204 (603/601)	0 (0%)	0 (0%)	n.a.
Severe pulmonary vein stenosis	4	683 (342/341)	1 (0.3%)	0 (0%)	1.43 (0.23–9.01)
Atrial flutter with 1:1 atrioventricular conduction	2	413 (206/207)	0 (0%)	3 (1.5%)	0.25 (0.03–2.25)
Ventricular tachycardia	6	1204 (603/601)	2 (0.3%)	3 (0.5%)	0.88 (0.22–3.46)
Bradycardia requiring pacemaker implantation	4	919 (464/455)	3 (0.6%)	5 (1.1%)	0.80 (0.21–3.05)
Syncope	5	1137 (571/566)	1 (0.2%)	8 (1.4%)	0.30 (0.08–1.12)

*AFCA, atrial fibrillation catheter ablation; AAD, anti-arrhythmic drug*.

* *This outcome was evaluated in the three most recent RCTs which used cryoenergy*.

§ *In all cases the palsy was reversible*.

## Discussion

The present meta-analysis indicates that, compared with AADs, first-line therapy with AFCA reduces arrhythmic recurrences in symptomatic patients with paroxysmal AF independently from the adopted energy source. This result is achieved without exposing the patients to an increase of adverse events. In addition, crossover to the alternative treatment arm is significantly less frequent in patients undergoing AFCA as first-line rhythm management. AFCA, in fact, is candidate gold standard treatment when sinus rhythm maintenance is strongly desired in symptomatic AF patients.

Periprocedural risk, mainly characterized by cerebral ischemic events, pericardial effusion (possibly leading to cardiac tamponade requiring urgent pericardiocentesis), and, particularly for cryoballoon ablation, phrenic nerve palsy is of concern. However, AFCA has dramatically improved its safety profile in the last decade. Technological advancement, increased experience, and shared evidence-based protocols (20), despite an increase in patient-specific risk profile (e.g., older patients in the “modern” cohort), have halved periprocedural complications in post-2010 compared with pre-2010 cohorts (2.3 vs. 5%) (21).

Conversely, AADs expose patients to a long-term risk of potential side effects, in particular pro-arrhythmic, which are, differently from AFCA, spread over time rather than concentrated in a specific period (22). The present analysis reports generally low complication rates in both study groups. However, the median 12-month follow-up may have limited collection of AAD-related side effects, while at the same time it likely captures all AFCA complications. Considering that patients initially treated with AADs, due to the overall modest efficacy of AADs in maintaining sinus rhythm (2), frequently undergo AFCA in the following 12 months (approximately one third), first-line AFCA may not only significantly shorten the “diagnosis-to-ablation time” maximizing rhythm outcome but also potentially prevent the sum of AFCA-related periprocedural risk and AADs side effects in drug-refractory patients.

Of note, the meta-analytic population was mainly constituted by relatively young, symptomatic patients, without overt underlying structural heart disease (guaranteed by the strict RCT inclusion criteria). As an example, median age in the CABANA (Catheter Ablation vs. Antiarrhythmic Drug Therapy on Mortality, Stroke, Bleeding, and Cardiac Arrest Among Patients With Atrial Fibrillation) trial (6) was significantly higher compared with the present population (67.5 vs. 55 years), with seven times more patients presenting with heart failure (15 vs. 2%) and higher thromboembolic risk (82% of patients with CHA2DS2-VASc score ≥2). Similarly, real-world data (23) indicate that on average AFCA candidates are older, with more comorbid conditions, higher thromboembolic risk profile, and frequent history of treatment failure with AADs. This suggests that particular caution should be used before extrapolating these data to the general AF population; however, it can be speculated that the observed low event rate of side effects in the AAD group might also relate to the healthier population. Unlike AFCA-related adverse events, which are mainly operator- and center-dependent, potential toxicity of AADs is known to increase with patient-related factors such as heart failure and underlying structural heart disease (22).

Finally, the present is the first analysis showing that cryoballoon ablation, previously demonstrating non-inferiority to RF ablation for the treatment of patients with drug-refractory paroxysmal AF (24), has similar efficacy to RF ablation also as a first-line option. In fact, cryoballoon ablation was reported to have fewer cardiac tamponades than ablation, and the most frequent and specific cryoballoon complication, phrenic nerve palsy, resolved within 1 month in three EARLY-AF (14) patients and was not present at 12-month follow-up in all STOP-AF cases (15); in Cryo-FIRST, the only transient case of phrenic nerve palsy occurred in a patient who crossed to AFCA arm after being randomized to AAD treatment arm (16).

### Limitations

Some limitations of the present analysis need to be addressed. First, the threshold defining an arrhythmic event varied between the included studies (from 15 to 60 s) and arrhythmia monitoring during follow-up was heterogeneous, ranging from clinical follow-up visits with intermittent rhythm monitoring to continuous monitoring with implantable loop records in one study (14). These differences may have an influence on the observed recurrent rate, even if a change in the proportional efficacy of the two treatment arms considered is unlikely. Second, also the type of arrhythmia considered as arrhythmic recurrences was variable between the studies, with two studies only including AF and four studies considering both AF and AT/AFL (Table 2), Nevertheless, arrhythmic recurrences are mainly AF episodes (25); thus, it is unlikely that the present results would have been affected by the inclusion of AT/AFL as arrhythmic recurrences in RAAFT-1 (10) and MANTRA-PAF (11). Third, we cannot exclude that the unblinded nature of the studies may have contributed to ablation benefit concerning symptomatic recurrences. Fourth, the analysis was not powered to detect potential significant differences in most of the secondary outcomes. Due to the low absolute number of events, we did not perform subgroup meta-analysis (RF or cryoenergy ablation) for the secondary outcomes.

## Conclusions

Compared with AADs, first-line therapy with AFCA, independently from the adopted energy source (RF or cryoenergy), reduces arrhythmic recurrences in symptomatic, paroxysmal AF patients without increasing exposure to complications. First-line AFCA, performed in high-volume experienced centers with low complication rate, candidates as for the preferable choice for sinus rhythm maintenance in relatively young, healthy patients without overt structural heart disease.

## Data Availability Statement

The original contributions presented in the study are included in the article/Supplementary Material, further inquiries can be directed to the corresponding author/s.

## Author Contributions

AS and MA conceived the study. AS performed literature search, data extraction, and statistical analysis. AS, FG, RD, GD, and MA critically analyzed the results and wrote the manuscript. All authors contributed to the article and approved the submitted version.

## Conflict of Interest

The authors declare that the research was conducted in the absence of any commercial or financial relationships that could be construed as a potential conflict of interest.
