# Supplementary material for: Catheter Ablation vs. Anti-Arrhythmic Drugs as First-Line Treatment in Symptomatic Paroxysmal Atrial Fibrillation: A Systematic Review and Meta-Analysis of Randomized Clinical Trials
Source: Front Cardiovasc Med. 2021 May 21;8:664647. doi: 10.3389/fcvm.2021.664647 (PMC8175669; doi:10.3389/fcvm.2021.664647)
Supplement: Supplementary file 1 [file Data_Sheet_1.docx]

**Catheter ablation versus anti-arrhythmic drugs as first-line treatment in symptomatic paroxysmal atrial fibrillation: a systematic review and meta-analysis of randomized clinical trials**

*Supplementary Material*

**Supplementary Table 1. Main inclusion and exclusion criteria for included studies.**

| **Study** | **Inclusion criteria** | **Exclusion criteria** |
| --- | --- | --- |
| RAAFT-1 (Wazni 2005) | Symptomatic AF for at least 3 months not treated by AADs | Age < 18 or > 75 years; previous AFCA; previous cardiac surgery; previous treatment with AADs;  contraindication to OAC treatment |
| MANTRA-PAF (Cosedis Nielsen 2012) | Symptomatic paroxysmal AF for at least 6 months; no episodes > 7 days; no previous or ongoing treatment with class IC or III AADs | Age > 70 years; previous or ongoing class IC or class III AADs; contraindication to class IC or class III AADs; previous AFCA; LA diameter > 5.0 cm; LVEF < 40%; contraindication to OAC; moderate-to-severe mitral valve disease; NYHA III-IV; expected surgery for structural heart disease; secondary AF |
| RAAFT-2 (Morillo 2014) | Symptomatic paroxysmal AF for at least 6 months not treated by AADs | Age < 18 or > 75 years; previous treatment with AADs; LVEF < 40%; LA diameter > 5.5 cm; left ventricular wall thickness > 1.5 cm; valve disease; coronary artery disease; previous cardiac surgery within 6 months; previous AFCA |
| EARLY-AF (Andrade 2020) | Symptomatic AF and at least one episode of AF detected on electrocardiography within 24 months before randomization | Age < 18 years; history of daily use of a class I or class III AAD; previous AFCA or LA surgery; contraindication to OAC; LVEF < 35%; LA diameter > 5.5 cm; moderate-to-severe mitral valve disease; cardiac valve prosthesis; hypertrophic cardiomyopathy; NHYA III-IV |
| STOP-AF (Wazni 2020) | Recurrent symptomatic paroxysmal AF | Age < 18 or > 80 years; previous treatment with AAD for > 7 days; previous AFCA or LA surgery; LA diameter > 5.0 cm; LVEF < 45%; NHYA III-IV |
| Cryo-FIRST (Kuniss 2021) | Recurrent symptomatic paroxysmal AF in drug naïve patients (had not previously received a  Class I or III AAD for >48 h); 18-75 years old; structurally normal heart with LVEF ≥ 50%, interventricular septum thickness ≤ 12 mm, and left atrial diameter < 46 mm; normal ECG parameters in sinus rhythm (QRS ≤ 120 ms, QTc interval < 440 ms, and PQ interval ≤ 210 ms) | At least one persistent AF episode; previous LA ablation; previous cardiac surgery including prosthetic valves; permanent pacemaker or defibrillator implant; documented typical atrial flutter; second degree type II or third degree AV-block or a pattern of left/right bundle branch block; history of previous myocardial infarction or percutaneous intervention during the last 3 months; history of stroke/TIA; NYHA > 1; hypertrophic cardiomyopathy; reversible cause of atrial fibrillation; channelopathy; COPD with pulmonary hypertensions; significant congenital heart defect corrected or not corrected; untreated or uncontrolled hyperthyroidism or hypothyroidism; glomerular filtration rate < 60 mL/min.; unstable angina pectoris; symptomatic carotid stenosis. |

AAD, antiarrhythmic drug; AFCA, atrial fibrillation catheter ablation; AF, atrial fibrillation; OAC, oral anticoagulation; LVEF, left ventricular ejection fraction; LA, left atrial.**Supplementary Table 2. Study-specific definitions of pre-specified primary and secondary endpoints.**

| **Study** | **Primary endpoint(s)** | **Secondary endpoint(s)** |
| --- | --- | --- |
| RAAFT-1 (Wazni 2005) | Recurrence of symptomatic or asymptomatic AF > 15 s during Holter or event monitoring | Hospitalization; QoL (SF-36) |
| MANTRA-PAF (Cosedis Nielsen 2012) | Recurrence of symptomatic or asymptomatic AF, atrial flutter or atrial tachycardia > 30 s on ECG or transtelephonic monitor | First documented recurrence and repeated episodes of symptomatic or asymptomatic AF, atrial flutter or atrial tachycardia; QoL (EQ-5D) |
| RAAFT-2 (Morillo 2014) | Percentage of time in AF on Holter recordings | Freedom from any AF; freedom from symptomatic AF; cumulative and per-visit burden of symptomatic AF; time to first recurrence of AF after the blanking period (3 months); atrial flutter longer than 1 min; QoL (SF-36) |
| EARLY-AF (Andrade 2020) | Recurrence of any atrial tachyarrhythmia (atrial fibrillation, atrial flutter, or atrial tachycardia) lasting 30 seconds or longer between 91 and 365 days | First recurrence of symptomatic atrial tachyarrhythmia between 91 and 365 days after the initiation of treatment; arrhythmia burden (percentage of time in atrial fibrillation); success of multiple ablation procedures; QoL; health care utilization; serious adverse events. |
| STOP-AF (Wazni 2020) | Primary efficacy endpoint Freedom from initial failure of the procedure, any subsequent atrial fibrillation surgery or ablation in the left atrium (including those performed during the blanking period), atrial arrhythmia recurrence (documented atrial fibrillation, atrial tachycardia, or atrial flutter for ≥30 seconds during ambulatory monitoring or for ≥10 seconds on a 12-lead ECG), cardioversion, or use of class I or III antiarrhythmic drugs (ablation group only) outside the 90-day blanking period  Primary safety endpoint (AFCA group only)  Composite of the following prespecified procedure-related or cryoballoon system–related serious adverse events: development of a clinically significant pericardial effusion within 30 days; symptomatic pulmonary vein stenosis or atrial–esophageal fistula within 12 months; unresolved phrenic nerve injury at 12 months; transient ischemic attack, stroke, myocardial infarction, major vascular complication, or major bleeding within the first 7 days | QoL (AFEQT, EQ-5D); health care utilization; serious adverse events; initial success of the procedure; procedural characteristics |
| Cryo-FIRST (Kuniss 2021) | Freedom from any atrial arrhythmia recurrence (at least one episode of AF, atrial flutter, or atrial tachycardia) lasting >30 s at 12 months documented by 7-day Holter ECG or any other ECG recording outside of the 90-day blanking period. | Serious adverse events; recurrence of patient-reported symptomatic palpitations. |

AFCA, atrial fibrillation catheter ablation; AF, atrial fibrillation; QoL, quality of life.

**Supplementary Figure 1. PRISMA Flow Chart.**

**
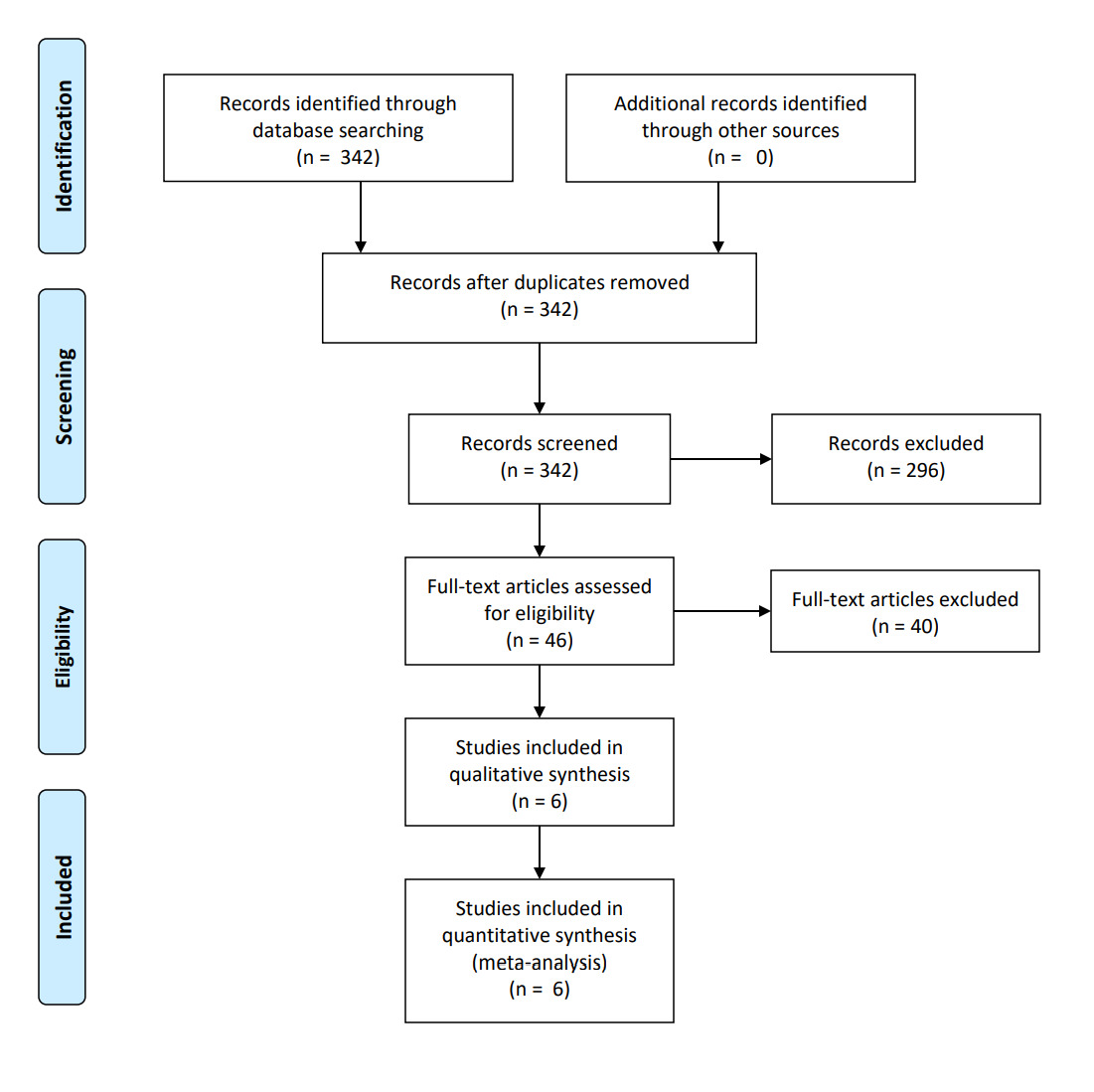
**

**Supplementary Figure 2. Forest plot reporting the risk of recurrence of atrial tachyarrhythmias (sensitivity analysis excluding RAAFT-1), stratified by ablation energy (radiofrequency or cryoenergy).**

**
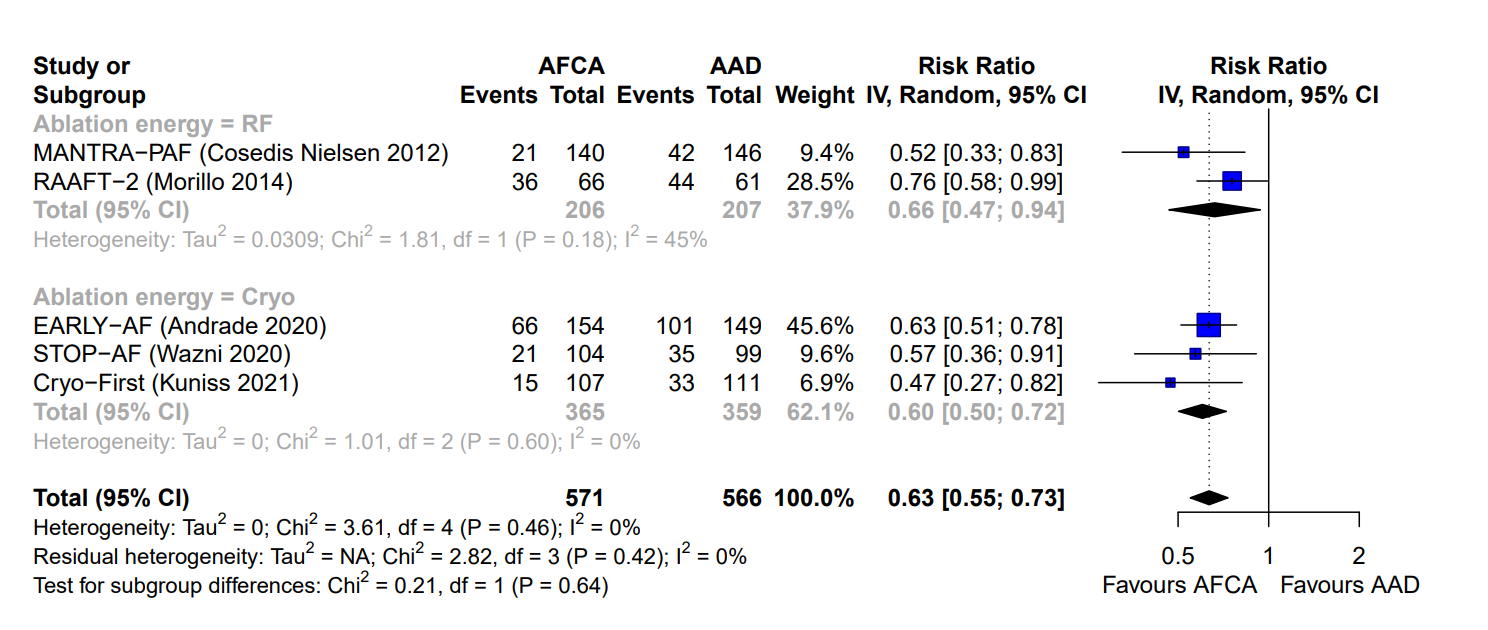
**

**Supplementary Figure 3.** **Forest plot reporting the risk of recurrence of symptomatic atrial tachyarrhythmias (sensitivity analysis excluding RAAFT-1), stratified by ablation energy (radiofrequency or cryoenergy).**

**
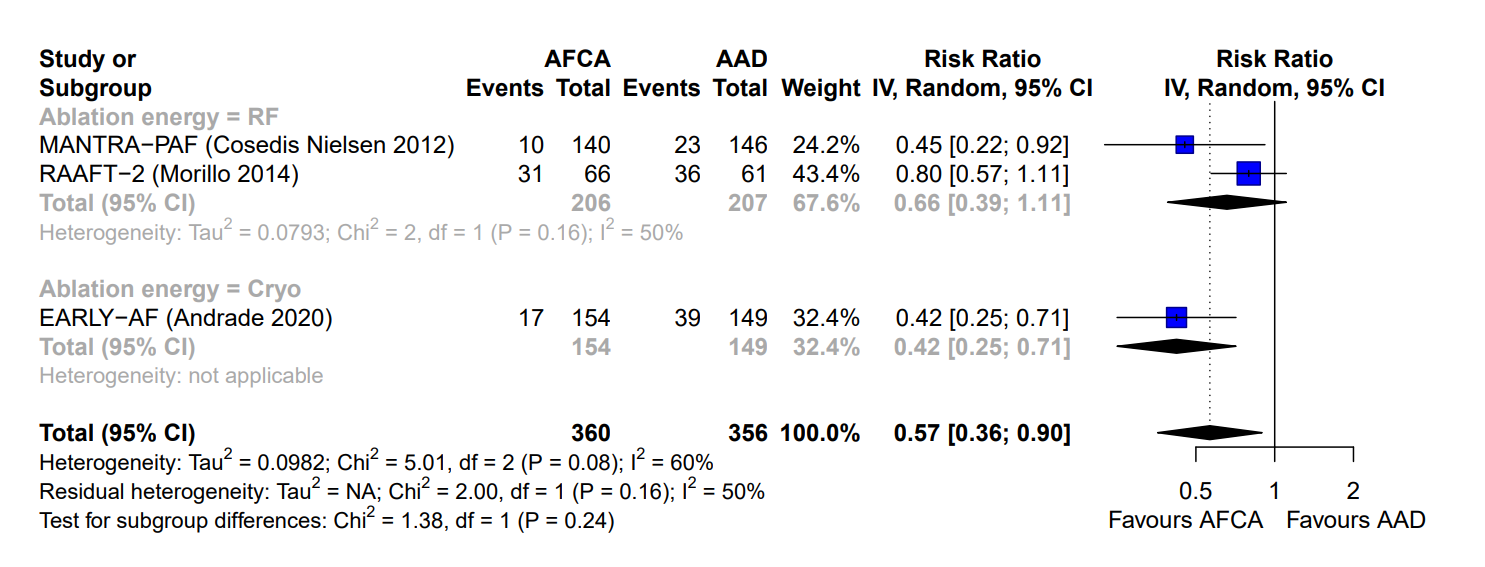
**

**Supplementary Figure 4. Forest plot reporting the risk of all-cause death.**

**
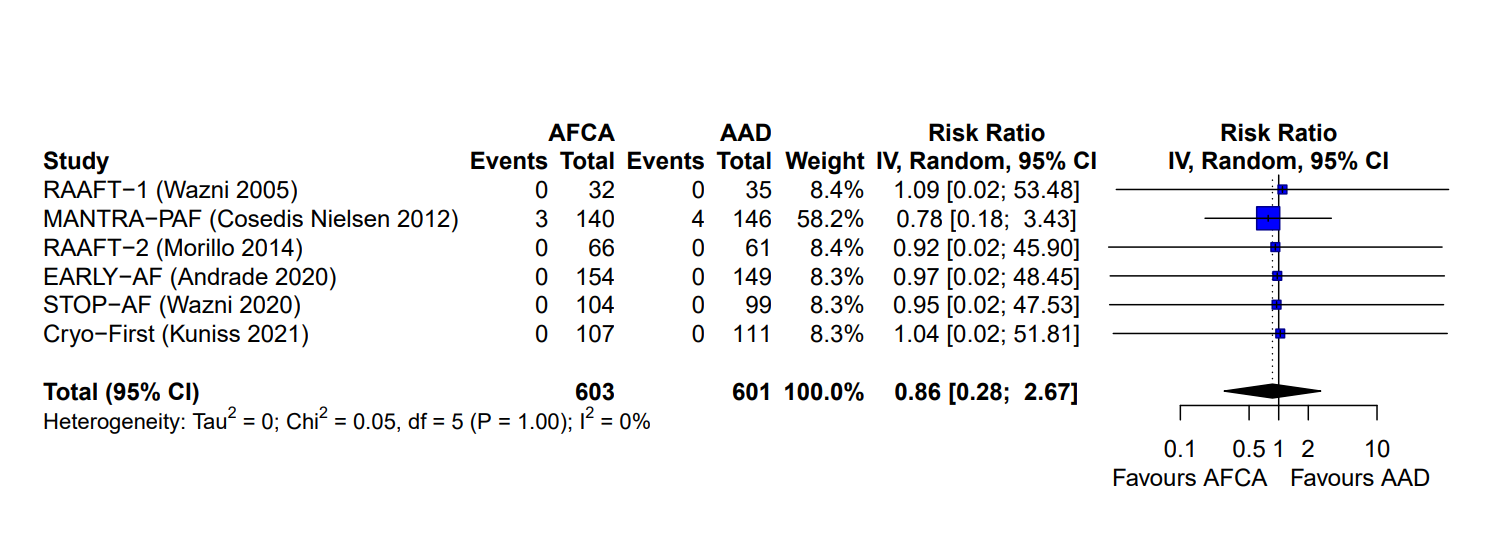
**

**Supplementary Figure 5. Forest plot reporting the risk of crossover to alternative treatment arm.**

**
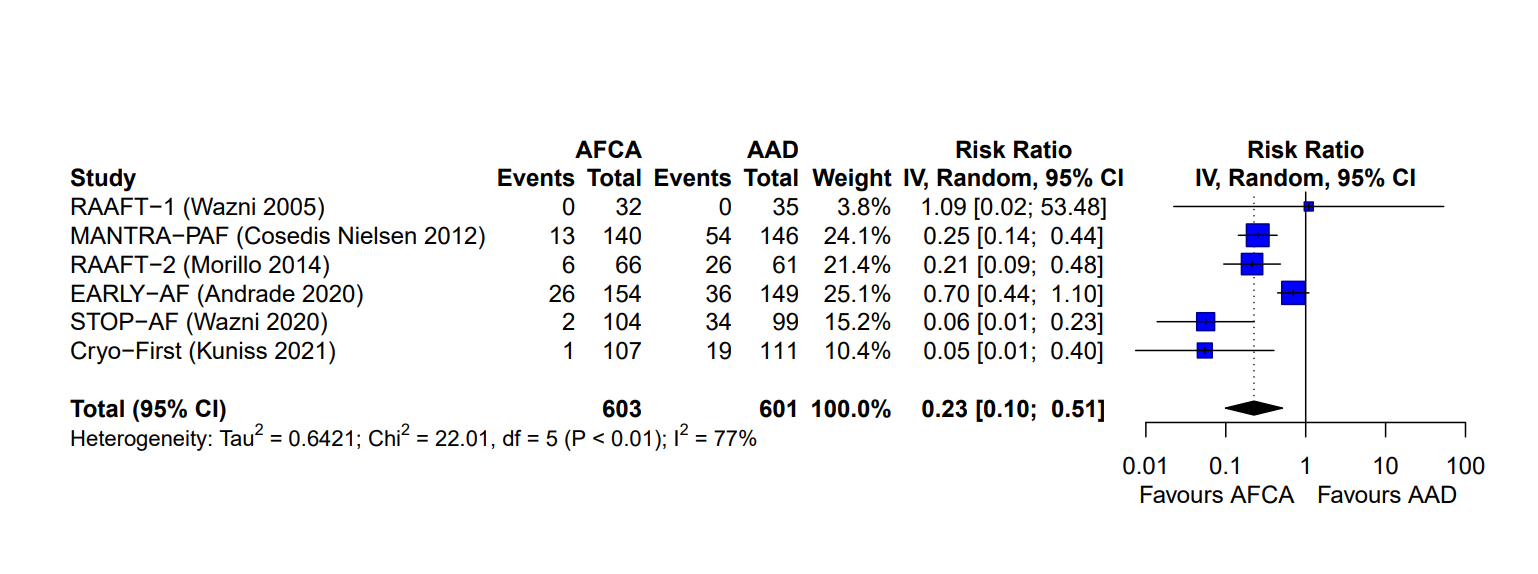
**

**Supplementary Figure 6. Forest plot reporting the risk of undergoing ablation during follow-up.**

**
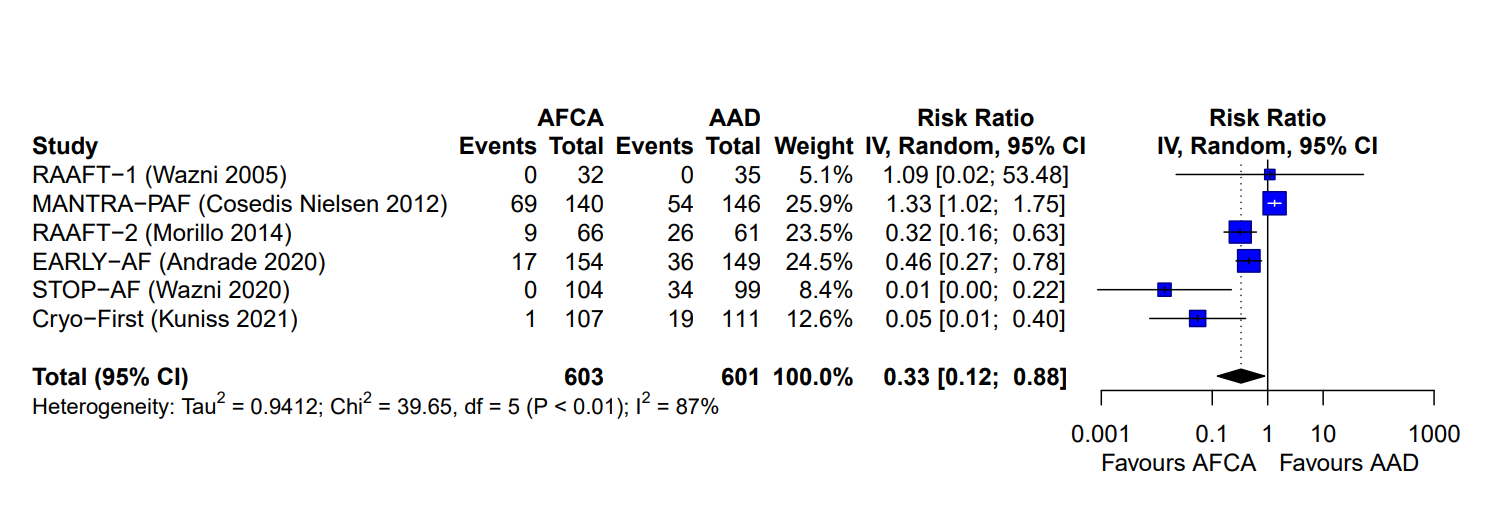
**

**Supplementary Figure 7. Forest plot reporting the risk of stroke/transient ischemic attack.**

**
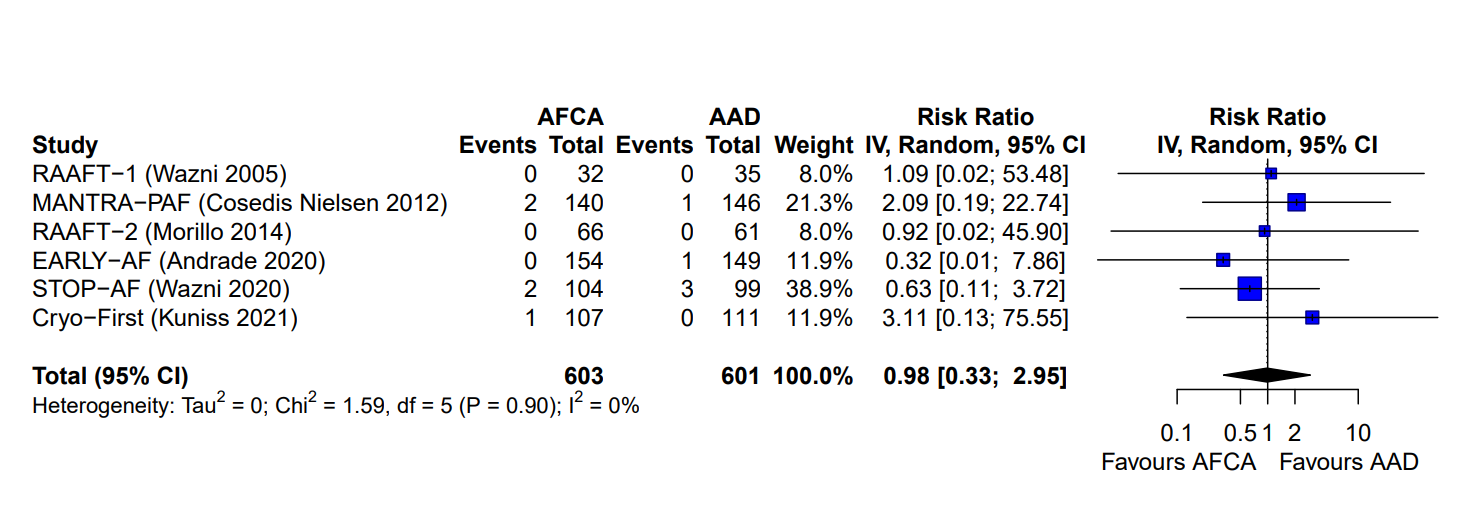
**

**Supplementary Figure 8. Forest plot reporting the risk of cardiac tamponade or clinically significant pericardial effusion.**

**
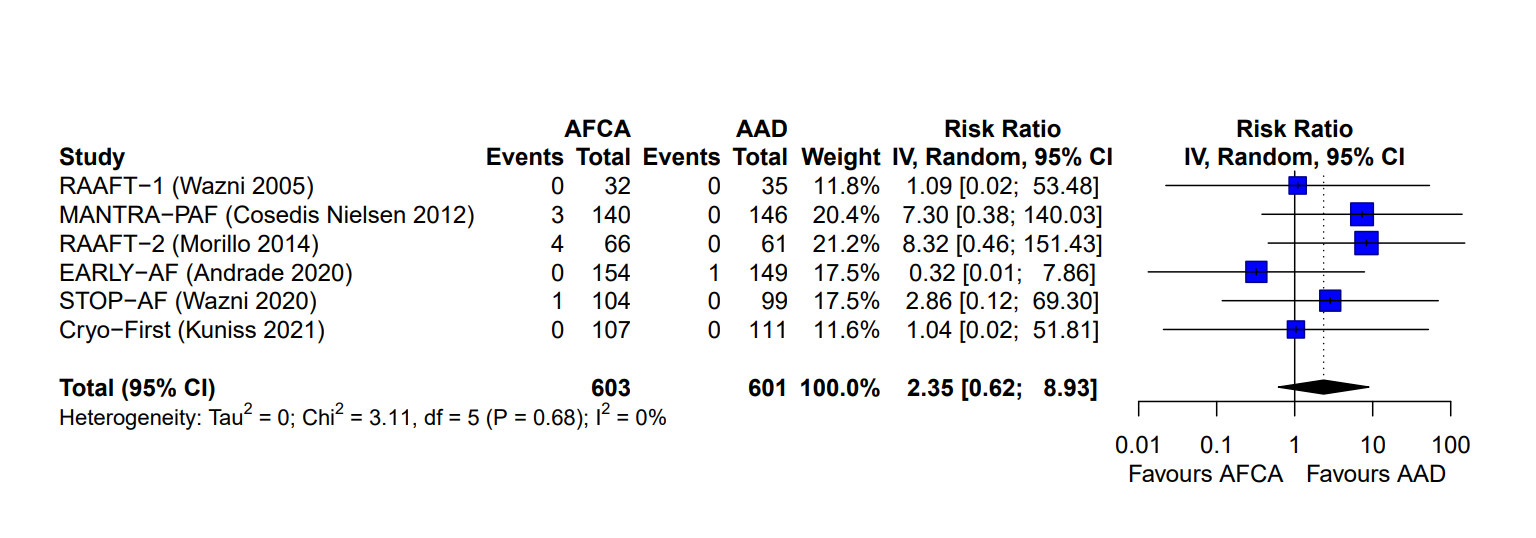
**

**Supplementary Figure 9. Forest plot reporting the risk of phrenic nerve palsy.**

**
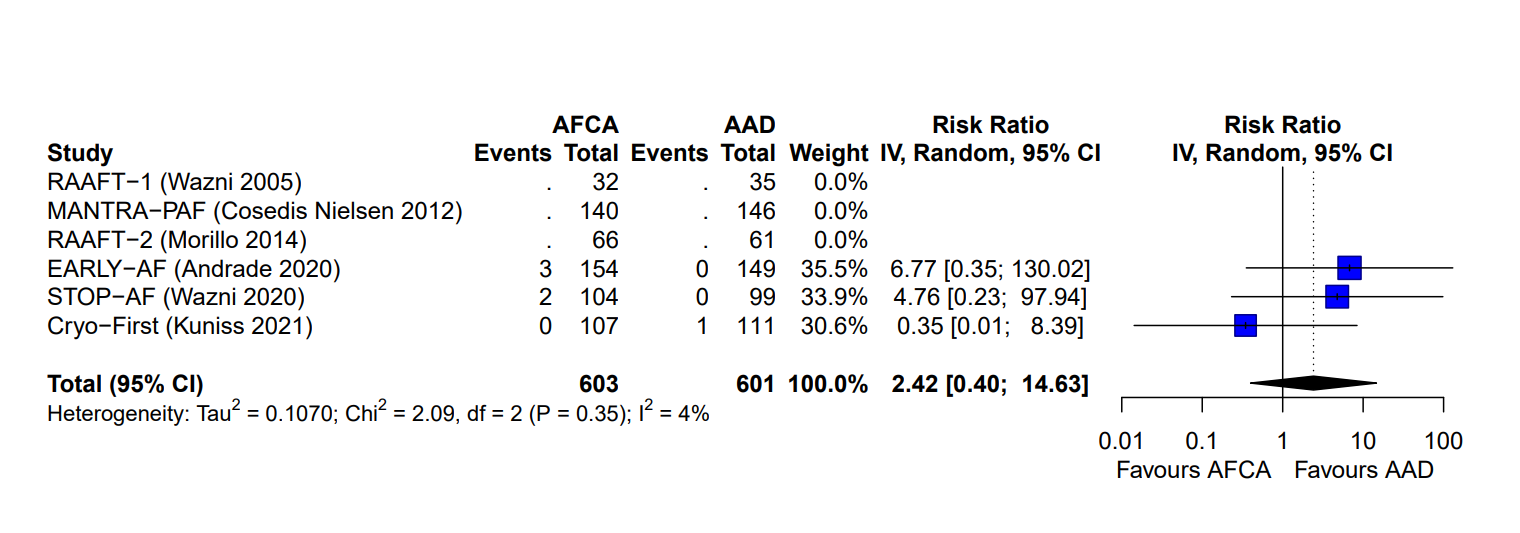
**

**Supplementary Figure 10. Forest plot reporting the risk of severe pulmonary vein stenosis.**

**
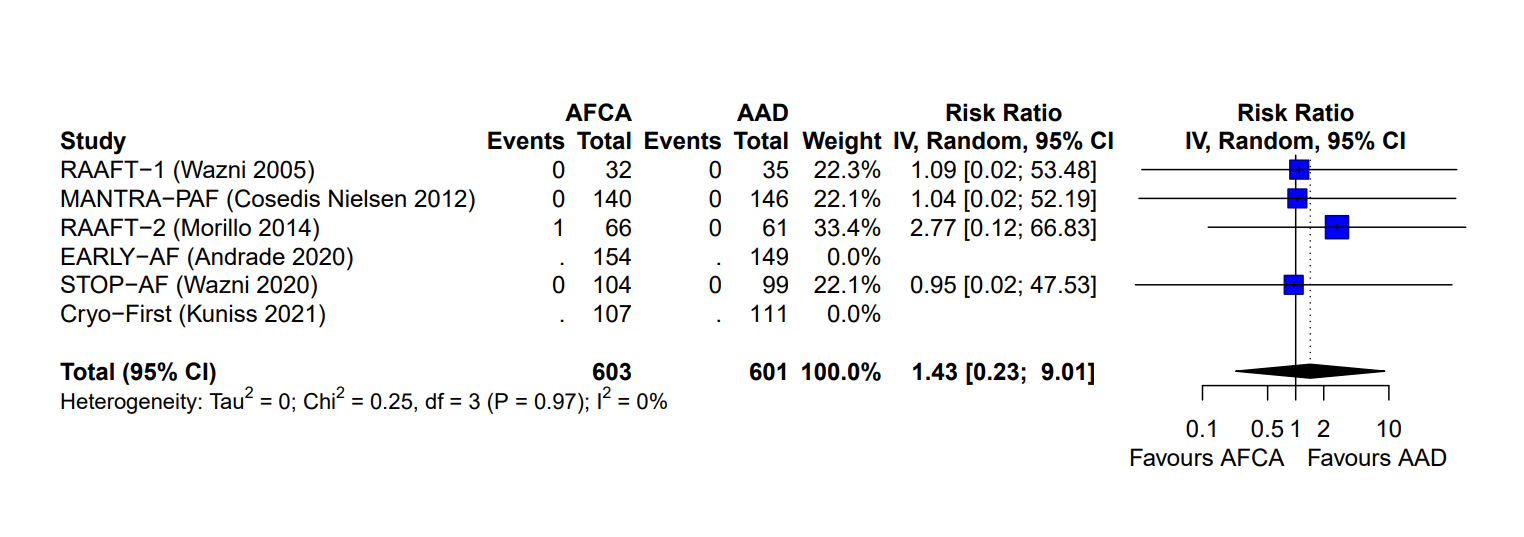
**

**Supplementary Figure 11. Forest plot reporting the risk of atrial flutter with 1:1 atrio-ventricular conduction.**

**
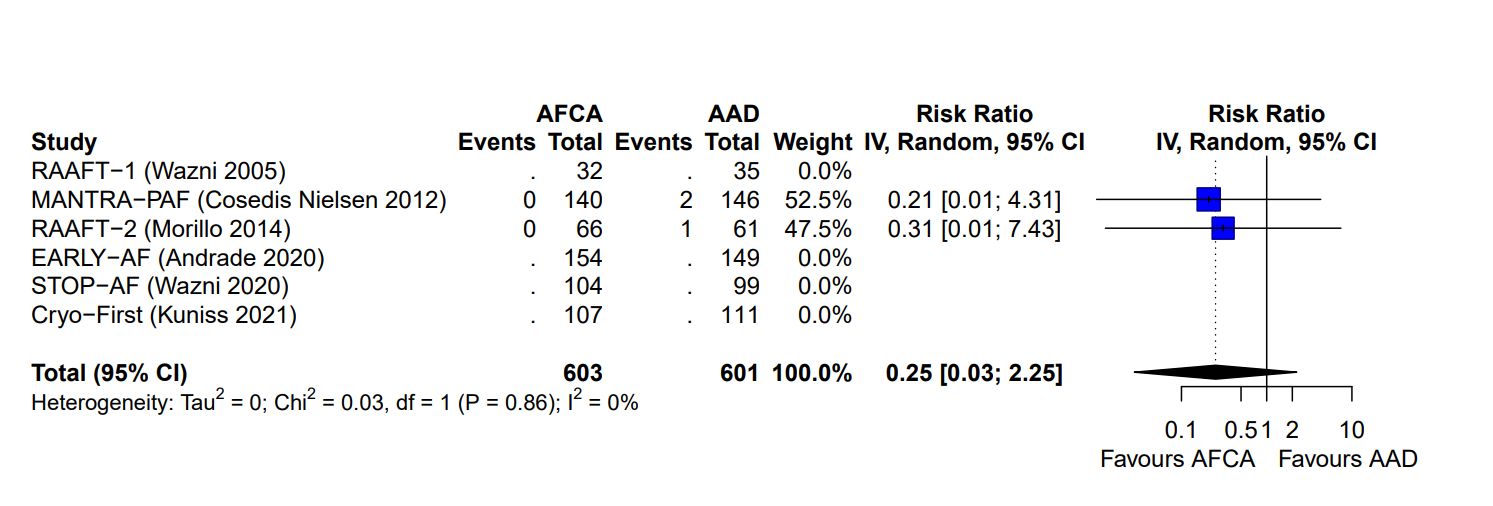
**

**Supplementary Figure 12. Forest plot reporting the risk of ventricular tachycardia.**

**
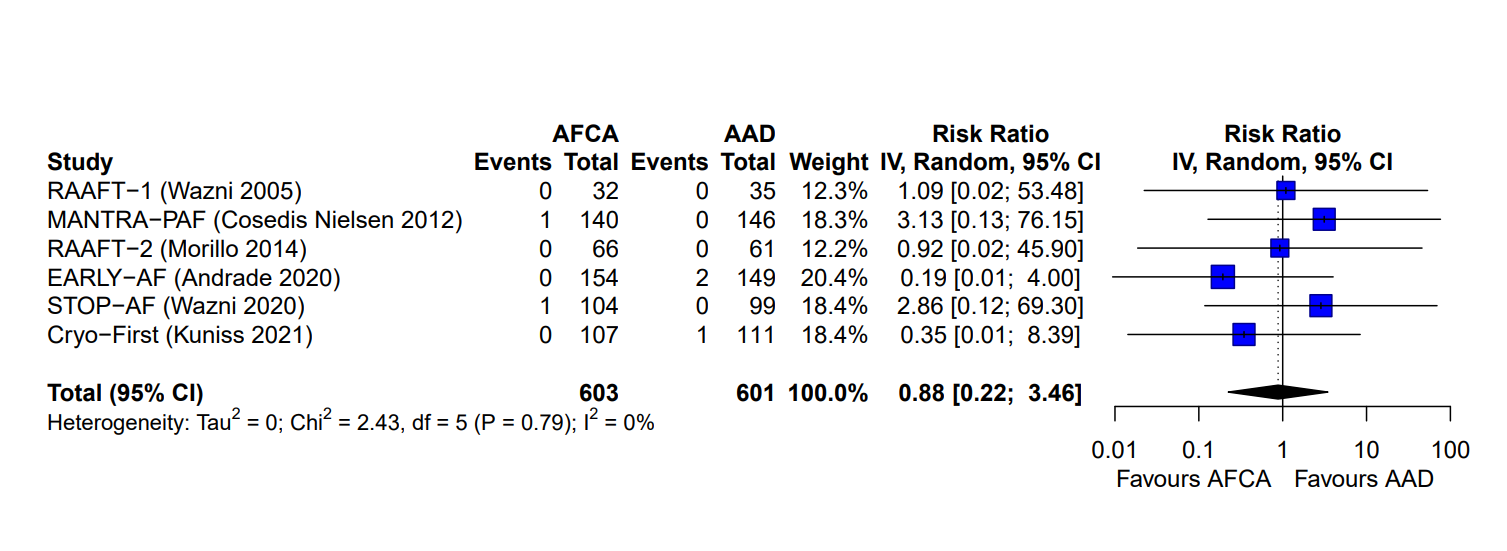
**

**Supplementary Figure 13. Forest plot reporting the risk of bradycardia requiring pacemaker implantation.**

**
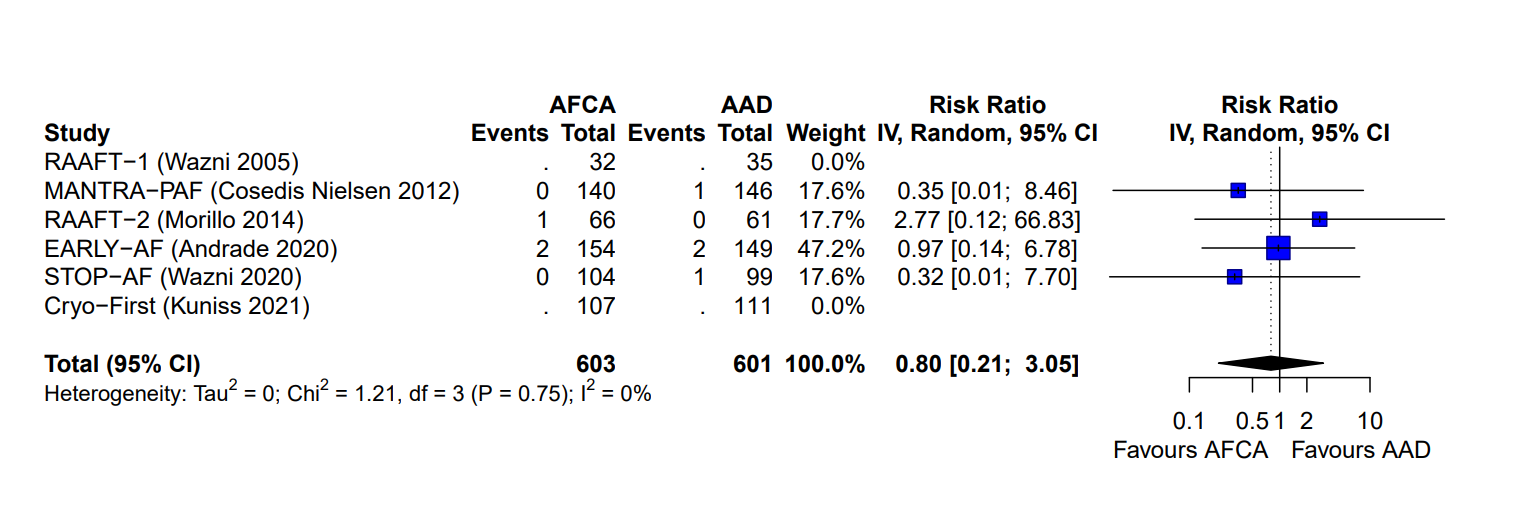
**

**Supplementary Figure 14. Forest plot reporting the risk of syncope.**

**
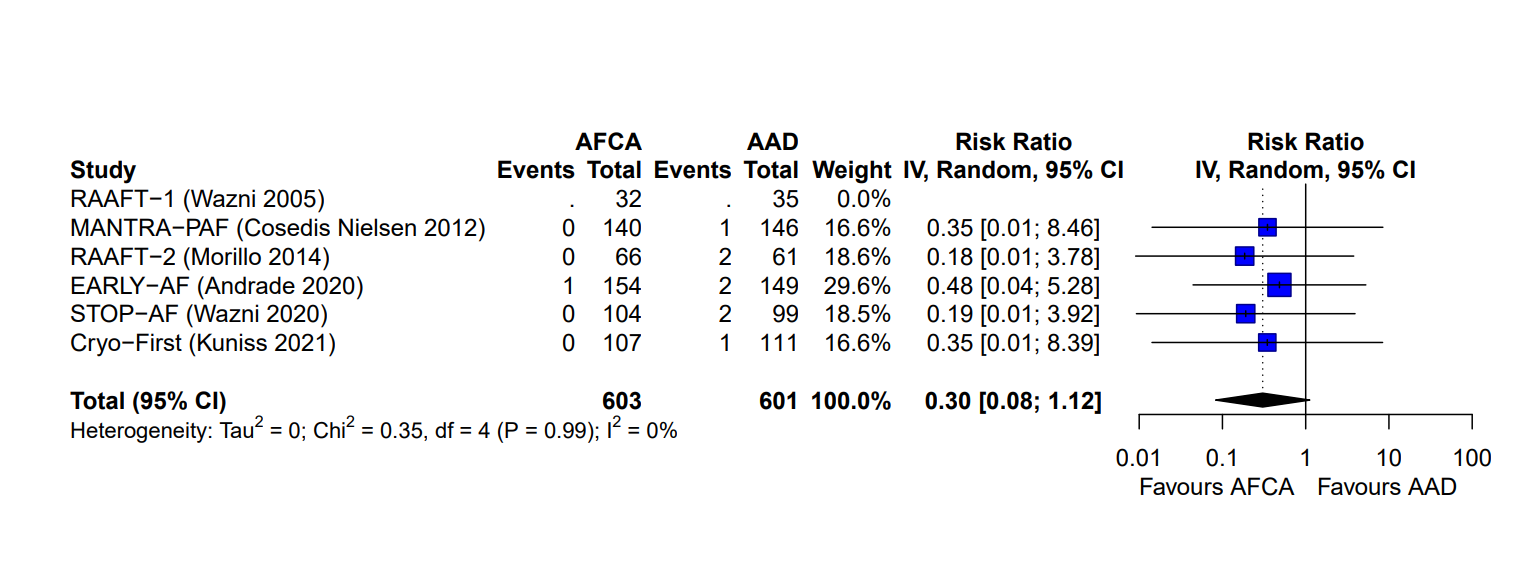
**
